# Southern Medical College Hospital

**Published:** 1881-02-20

**Authors:** 


### *SOUTHERN MEDICAL COLLEGE HOSPITAL.*

It will interest the friends of medical education in the South to learn that ere long all the needed facilities for securing a medical education of the highest order will be found in Atlanta.

The Trustees and Faculty of the Southern Medical College have determined to supply the only want which they have heretofore felt was necessary to place the Institution second to no school in the United States in respect to educational facilities, and that is a hospital.

Steps have already been taken to accomplish this desired object. A central and eligible lot has been secured, upon which is a building of good size. To this, improvements and commodious additions will be made, and the entire structure will be ready for occupants by the opening of the next course of lectures in the Institution.

---

*Proceedings of the Louisiana State Medical Society*, at its third meeting held in the city of New Orleans, March 31st, 1880.

The contents consist of the following interesting papers:

President's Annual Address—Dr. J. W. Dupree; Address of Annual Orator—Rev. H. M. Thompson, D. D.; Report of Standing Committee on State Medicine—Prof. S. E. Chaille, M. D.; Report of Chairman of Auxiliary Committee on State Medicine—Dr. J. P. Davidson; Report on Public Hygiene—Dr. S. S. Herrick; Report on Medical Education—Dr. C. J. Bickham; Report of the Corresponding Secretary—Dr. S. S. Herrick; Report on Hydrophobia—Prof. T. G. Richardson, M. D.; The Conservative Influence of Diseases—Dr. A B. Small; Medical History of Plaquemines Parish, 1879—Dr. D. B. Fox; Incised Wounds of the Abdomen—Dr. R. H. Day; Cold Douche in Algid Fever—Dr. A. W. DeRoaldes; Morbus Coxarius—Dr. M. Schuppert.

The following are the officers elect for the year, to-wit:

*President*—Dr. C. M. Smith, Franklin, St. Mary Parish; *Vice-Presidents*—Dr. D. R. Fox, First Congressional District, Jesuit's Bend, Plaquimines Parish; Dr. J. P. Davidson, Second Congressional District, No. 473 Carondelet Street, New Orleans; Dr. P. S. Postell, Third Congressional District, Plaquemine, Iberville Parish; Dr. A. A. Lyon, Fourth Congressional District, Shreveport; Dr. G. M. Brumby, Fifth Congressional District, Delhi, Richmond Parish; Dr. O. P. Langworthy,

Sixth Congressional District, Clinton, East Feliciana Parish; *Recording Secretary*—Dr. L. F. Solomon, No. 19 Baronne Street, New Orleans; *Corresponding Secretary*—Dr. S. S. Herrick, No. 427 Carondelet Street, New Orleans; *Treasurer*—Dr. Geo. K. Pratt, Corner Canal and St. Charles Street, New Orleans.

